# Case Report: Transit bipartition: early postoperative food tolerance and bowel function

**DOI:** 10.3389/fnut.2026.1759391

**Published:** 2026-03-17

**Authors:** Carina Rossoni, Rui Ribeiro

**Affiliations:** 1 Faculty of Medicine, Institute of Environmental Health, University of Lisbon, Lisbon, Portugal; 2 Multidisciplinary Center for the Treatment of Obesity and Diabetes at Lusíadas Hospital Amadora, Amadora, Portugal; 3 Multidisciplinary Center for Treatment of Obesity and Diabetes at Lusíadas Hospital Lisbon, Lisbon, Portugal

**Keywords:** bariatric and metabolic surgery, bowel function, dietary progression, food tolerance, gastric bipartition and transit bipartition

## Abstract

**Introduction:**

We report a case demonstrating excellent food tolerance and preserved intestinal function 30 days after transit bipartition, following implementation of an early, structured, and differentiated postoperative nutritional protocol.

**Case presentation:**

A 65-year-old Caucasian woman with a body mass index (BMI) of 57.7 kg/m^2^ presented with a long-standing history of obesity beginning in childhood, a positive family history, and symptom exacerbation during her first of two pregnancies. Comorbidities included functional thrombocytopenia (von Willebrand disease related to factor X deficiency), depression, anxiety, obstructive sleep apnea syndrome (OSAS) requiring continuous positive airway pressure (CPAP) therapy, degenerative osteoarticular disease, and dyslipidemia. Eating behavior assessment revealed emotional eating, binge eating disorder, and volume eating. Dietary intake was characterized by excessive consumption of carbohydrates and sweets (particularly bread), with insufficient intake of fruits, vegetables, and dairy products. The patient underwent laparoscopic transit bipartition, with construction of a 250 cm common limb and a 50 cm ileal bridge. Postoperative nutritional management adhered to enhanced recovery after surgery for bariatric surgery (ERAS-BS) principles. Dietary progression was structured according to the International Dysphagia Diet Standardization Initiative (IDDSI) framework. Food tolerance was evaluated using the validated food quality and tolerance questionnaire proposed by Suter et al, and bowel function was assessed using the Bristol Stool Scale.

**Discussion:**

Thirty days after surgery, the patient demonstrated excellent alimentary tolerance, achieving a score of 21 on the Suter questionnaire. She reported no nausea, vomiting, or other gastrointestinal symptoms. Stool consistency corresponded to types 3–4 on the Bristol Stool Scale, indicating normal bowel function. The prescribed protein supplementation target of 25 g/day was achieved and well tolerated. During this period, the patient experienced a total weight reduction of 12.2 kg (6.4 kg of fat mass) accompanied by decreases of 5 cm and 8 cm in neck and abdominal circumference, respectively.

**Conclusion:**

The proposed nutritional protocol—characterized by early dietary introduction, structured weekly progression in food consistency, and systematic protein, vitamin, and mineral supplementation—proved to be safe and effective. This approach facilitated excellent food tolerance and normal intestinal function, with no gastrointestinal adverse effects observed during the early postoperative period following transit bipartition.

## Introduction

**Figure 1:**
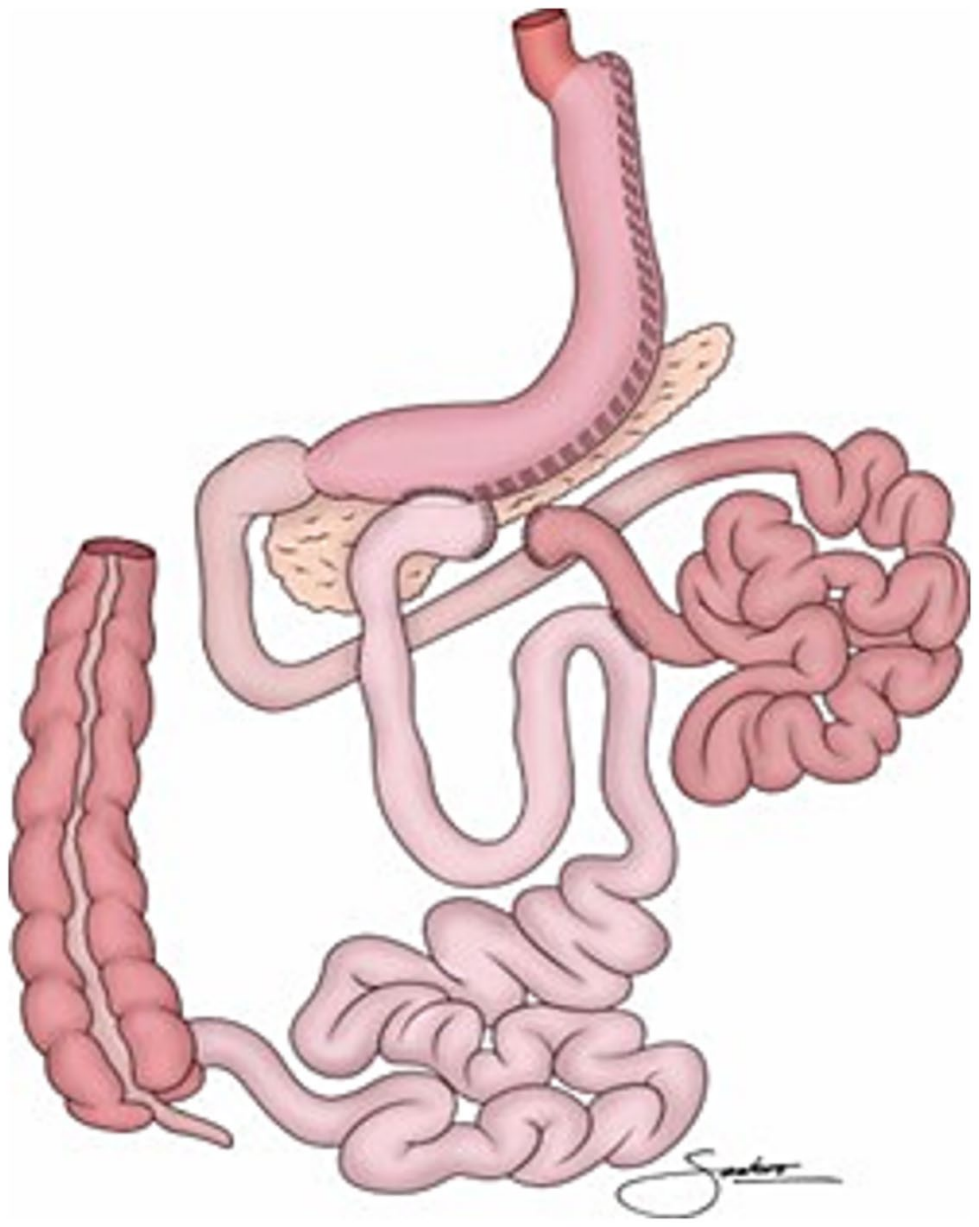
Transit bipartition, adapted from Santoro et al. (1), licensed under CC BY 4.0.

Transit bipartition is an ileal surgical technique developed by Santoro et al. (1) (Figure 1). It is characterized by functional restrictions, i.e., a metabolic delay of gastric emptying and intestinal transit speed, rather than physical restriction (1, 2). Physiologically, increased production of GLP1 induces satiety, while a gastro-ileostomy promotes the early arrival of gastric contents in the ileum. This corrects proximal bowel hyperactivity and ileal hypoactivity, reducing GIP secretion and stimulating the production of GLP1, PYY, OXM and FGF-19. These mechanisms promote insulin sensitivity, type 2 diabetes remission, weight reduction and nutritional stability, while preserving the presence of food in the duodenum and jejunum and ensuring endoscopic access to the entire gastrointestinal system (1–3).

According to the ERAS-BS protocol (4), one of the primary objectives of enhanced recovery programs is to achieve early nutritional stability. Nutritional management is integrated into three of the four phases of the protocol: pre-admission, preoperative, and postoperative. In the postoperative phase, early oral feeding should be initiated alongside appropriate vitamin and mineral supplementation (4).

However, in clinical practice, there remains a persistent stigma and resistance toward early nutritional progression and protein supplementation. This approach may result in unnecessarily prolonged periods of overly restrictive and nutritionally inadequate intake, potentially compromising metabolic recovery, psychological well-being, and anthropometric outcomes. Furthermore, such restrictions may delay the development of nutrition education, healthy eating behaviors, and sustainable lifestyle modifications—which are central goals of nutritional management in bariatric surgery.

Given the absence of published data specifically addressing nutritional intervention after transit bipartition, we aimed to evaluate the outcomes of a differentiated postoperative nutritional protocol characterized by early initiation and structured weekly progression of diet consistency. By assessing food tolerance and bowel function during the first 30 postoperative days, we demonstrate that this early and progressive protocol is safe and well tolerated.

## Case report

### Patient information

A 65-year-old Caucasian woman with a history of childhood-onset obesity, a positive family history of obesity, and symptom exacerbation during the first of two pregnancies was evaluated. Her comorbidities included functional thrombocytopenia (von Willebrand disease related to factor X deficiency), depression, anxiety, obstructive sleep apnea syndrome (OSAS) requiring continuous positive airway pressure (CPAP) therapy, degenerative osteoarticular disease, and dyslipidemia. Her regular medications included escitalopram 20 mg/day, bupropion 150 mg/day, and lamotrigine 100 mg/day. She had no history of nephrolithiasis, was a non-smoker, and reported regular bowel habits.

### Nutritional assessment and diagnosis

Baseline anthropometric assessment showed a body weight of 157 kg, height of 165 cm, and body mass index (BMI) of 57.7 kg/m^2^. Neck and abdominal circumferences were 50 cm and 158 cm, respectively. Body composition analysis by bioelectrical impedance (Tanita^®^ DC430-S MA) revealed a fat mass of 92.9 kg, corresponding to 59.2% of total body weight (Table 1), consistent with class IV obesity and a markedly increased cardiometabolic risk profile. The patient reported a sedentary lifestyle.

**Table 1:** Anthropometric and body composition parameters.

Parameters	Preop.	12 days PO	30 days PO
Anthropometric			
Height (cm)	165.0		
Weight (kg)	157.0	150.0	145.7
BMI (kg/m^2^)	57.7	55.09	53.5
Neck circ. (cm)	50.0	—	42.0
Abdominal circ. (cm)	158.0	—	150.0
Body composition			
Body fat (kg)	92.9	—	86.5
Body fat (%)	59.2	—	59.4

Dietary assessment demonstrated excessive carbohydrate intake, particularly bread and sweets, with insufficient consumption of fruits, vegetables, and dairy products. She reported no food allergies or intolerances. Eating behavior was characterized by emotional eating, compulsive eating, and overeating.

Biochemical evaluation identified vitamin D deficiency. Preoperative vitamin D supplementation was initiated, together with pharmacological treatment for dyslipidemia and eradication therapy for *Helicobacter pylori*, according to medical prescription.

### Nutritional intervention

The pre-admission nutritional protocol was initiated 4 months before surgery with the aim of reducing surgical risk. It focused on nutritional education emphasizing dietary quality, correction of identified nutritional deficiencies through supplementation, reduction of body weight and liver volume, and minimization of the preoperative fasting period.

The nutritional intervention was defined following assessment and diagnosis and consisted initially of a norm consistent, hypocaloric diet (1,200 kcal/day) (5). The dietary plan emphasized protein-rich foods, complex carbohydrates, soluble and insoluble fiber, and unsaturated fatty acids, together with increased water intake and reduced consumption of sweets. During the second and third consultations, the meal plan was maintained, and healthy eating behaviors were reinforced. At the fourth consultation, a preoperative nutritional protocol was instituted, consisting of a liquid very low-calorie diet (6) (800 kcal/day providing 70 g of protein per day) for the 10 days preceding surgery, combined with protein, vitamin, and mineral supplementation. The primary objective was reduction of liver volume.

In accordance with the principles of the ERAS-BS protocol (2021) (4), preoperative fasting was shortened to three hours before the procedure. For this purpose, the oral nutritional supplement *Fresubin Jucy Drink^®^* was administered. This formulation contains 11% protein (whey) and 89% carbohydrates (maltodextrin and sucrose), with no lipid content. The supplement is free of fiber, lactose, and gluten.

**Figure 2:**
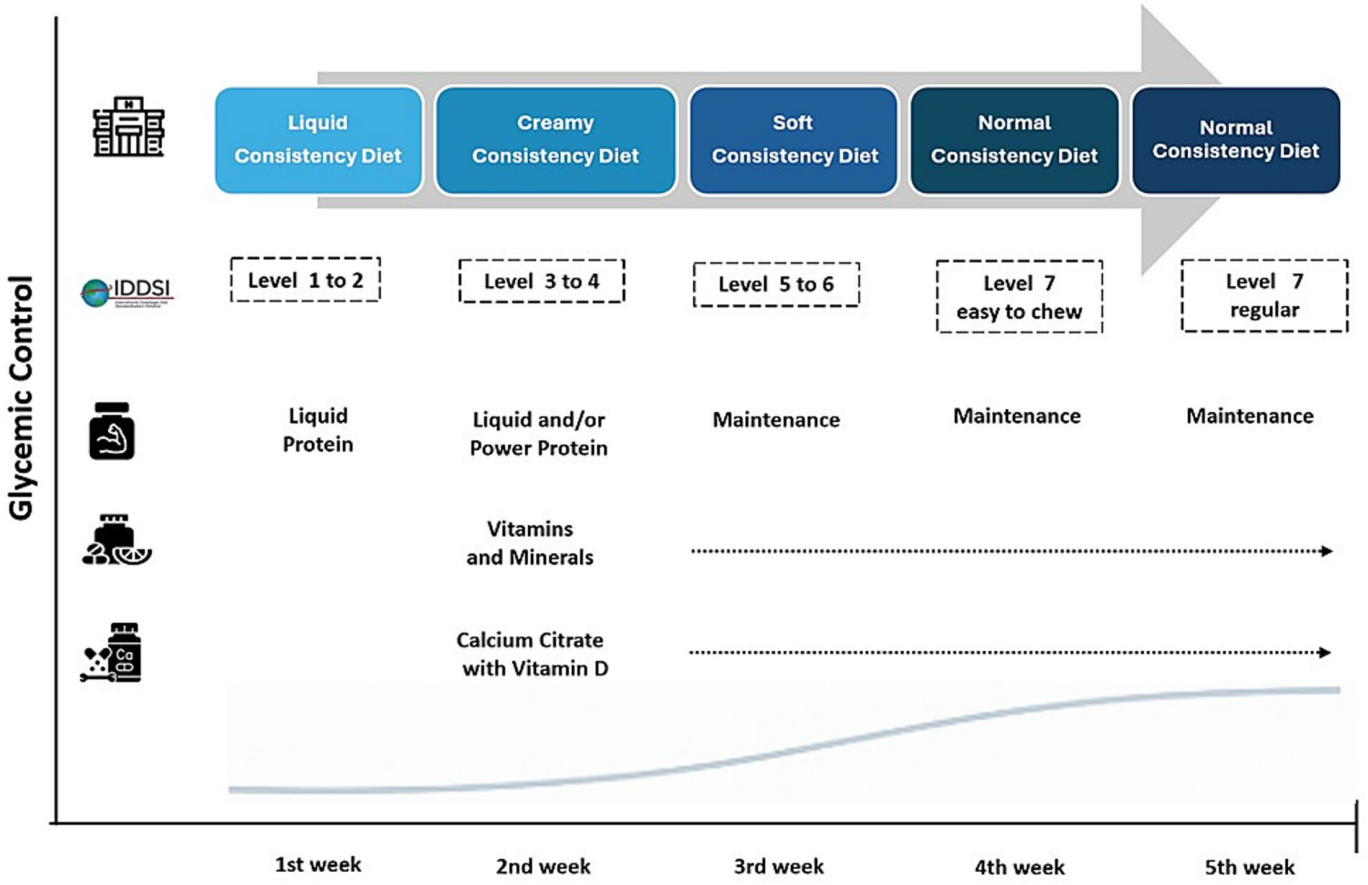
Nutritional protocol—progression of diet consistency and nutritional supplementation during the early postoperative period.

The patient underwent a classic transit bipartition (Santoro procedure), consisting of a vertical sleeve gastrectomy (SG) combined with a gastro-ileostomy. The common limb measured 250 cm from the ileocecal valve, and the ileal bridge 50 cm. The postoperative nutritional protocol (Figure 2) (7–9) was initiated early, 6 h after surgery, and consisted of a liquid diet including water, tea, and liquid protein supplementation (≥20 g protein). Intake was initiated at 20 mL every 30 min during the first 24 h postoperatively. Other key components of immediate postoperative care included early ambulation, pharmacological thromboprophylaxis, and assisted physical therapy.

After hospital discharge, natural fruit juices and vegetable broth were introduced on postoperative day 1, with a gradual increase in volume and extension of the intervals between meals. By the end of the first week, intake had progressed to 80 mL every 45 min, providing approximately 520 kcal/day and a mean protein intake of 45 g/day, in addition to supplemental water consumption. During the second postoperative week, creamy diet was initiated, providing an average of 570 kcal/day and 61 g of protein per day. This phase included blended vegetable soups with lean meat, poultry, fish, or legumes; cooked or baked fruits; thick, non-acidic fruit juices; and an expanded selection of dairy products, with or without lactose.

Protein supplementation was prescribed with a target intake of 25 g/day, using unflavored protein powder (e.g., Fresubin^®^ Protein Powder, Resource^®^ Instant Protein, or Protifar^®^) and/or specialized liquid protein formulations for surgical patients, including immunonutrition (Impact^®^). Vitamin and mineral supplementation was initiated on postoperative day 8 and consisted of WLS Primo^®^ (one capsule daily) and Calcium Soft Chew^®^ (two units daily).

### Follow-up and outcomes

The first postoperative nutritional consultation occurred on postoperative day 12. The patient reported no episodes of nausea or vomiting, regular bowel movements, and a daily fluid intake of approximately 1,500 mL. She reported good tolerance of the prescribed diet during the first two postoperative weeks, with no diet-related complications.

Following nutritional assessment and diagnosis at this consultation, the intervention consisted of progression to a third-week soft diet, providing an average of 710 kcal/day and 65 g of protein per day. At this stage, all lean meats were required to be shredded, minced, or ground; legumes were thoroughly cooked; and vegetables were permitted in puréed form.

During the fourth postoperative week, the patient progressed to a normal-consistency diet, providing an average of 830 kcal/day and 70 g of protein per day, corresponding to 30 days of postoperative care. Foods were prepared by cooking, grilling, and/or baking and were served in pieces that were easy to chew. Notably, the staged progression of diet consistency followed the IDDSI protocol levels (7–9).

Thirty days after surgery, food tolerance and dietary quality were assessed using the instrument proposed by Suter et al. (10). This tool evaluates:

1. Patient satisfaction with overall diet quality.
2. Meal duration and food intake between meals.
3. Tolerance to eight different food categories.
4. Frequency of vomiting or regurgitation following bariatric and metabolic surgery.

The total score is calculated from components 1, 3, and 4, with possible values ranging from 1 to 27, where higher scores indicate better food tolerance. In this clinical case, the final score was 21, corresponding to excellent food tolerance. This result should be interpreted in the context of the first 30 postoperative days, during which raw vegetables, pasta, and bread are intentionally excluded from the nutritional protocol. The patient reported no episodes of nausea or vomiting and maintained a daily water intake of approximately 1,500 mL.

Bowel function was assessed using the Bristol Stool Scale (11). The patient reported daily bowel movements with stool consistency corresponding to types 3–4, which are considered healthy/ideal. She reported only rare occurrences of type 2 stools.

Anthropometric and body composition assessment demonstrated a reduction in body weight of 12.2 kg, of which 6.4 kg was attributable to fat mass loss. Neck and waist circumferences decreased by 5 cm and 8 cm, respectively (Table 1).

At postoperative day 30, the nutritional intervention aimed to reinforce adherence to protein, multivitamin, and calcium supplementation and to advance the patient to a normal-consistency diet, including raw foods, providing an average intake of 1,000 kcal/day and 80 g of protein per day. Sweets, fried foods, carbonated beverages, and alcoholic drinks were contraindicated. This nutritional intervention was combined with supervised resistance-training exercises.

This case report was conducted in accordance with the ethical principles outlined in the Declaration of Helsinki (1964) and its subsequent amendments, as well as with applicable ethical standards. Written informed consent was obtained from the patient after detailed explanation of the study, and the report was submitted to the Ethics Committee of Hospital Lusíadas Amadora, which issued a favorable opinion.

## Discussion

According to major international guidelines for bariatric and metabolic surgery, patients should begin with a liquid diet and progressively advance to puréed, soft, and normal-consistency foods, based on individual tolerance (4, 5, 12). However, the optimal timing and structure of dietary progression after bariatric and metabolic surgery remain subjects of ongoing debate.

In oncological procedures such as total gastrectomy, evidence has demonstrated that early postoperative dietary initiation with gradual progression of food consistency is safe (13). In contrast, evidence supporting comparable postoperative nutritional strategies in ileal procedures—such as transit bipartition (TB), single-anastomosis duodeno-ileal bypass with sleeve gastrectomy (SADI-S), one-anastomosis gastric bypass (OAGB), and ileal interposition (II)—remains limited.

In our clinical practice, transit bipartition has been associated with superior food tolerance, as illustrated by the results of this clinical case, when compared with sleeve gastrectomy or Roux-en-Y gastric bypass, procedures characterized by more pronounced mechanical restriction. In this context, the effective evaluation of differentiated postoperative nutritional protocols using validated and widely accepted assessment tools is essential, as demonstrated in the present case.

We acknowledge the need for prospective studies, including randomized controlled trials (RCTs), to establish the safety of this differentiated nutritional protocol across different bariatric and metabolic surgical techniques. Previous studies assessing food tolerance during the first postoperative month have been conducted in patients undergoing sleeve gastrectomy, Roux-en-Y gastric bypass, Fobi-Capella gastric bypass, one-anastomosis gastric bypass, and biliopancreatic diversion with or without duodenal switch (14–16).

**Figure 3:**
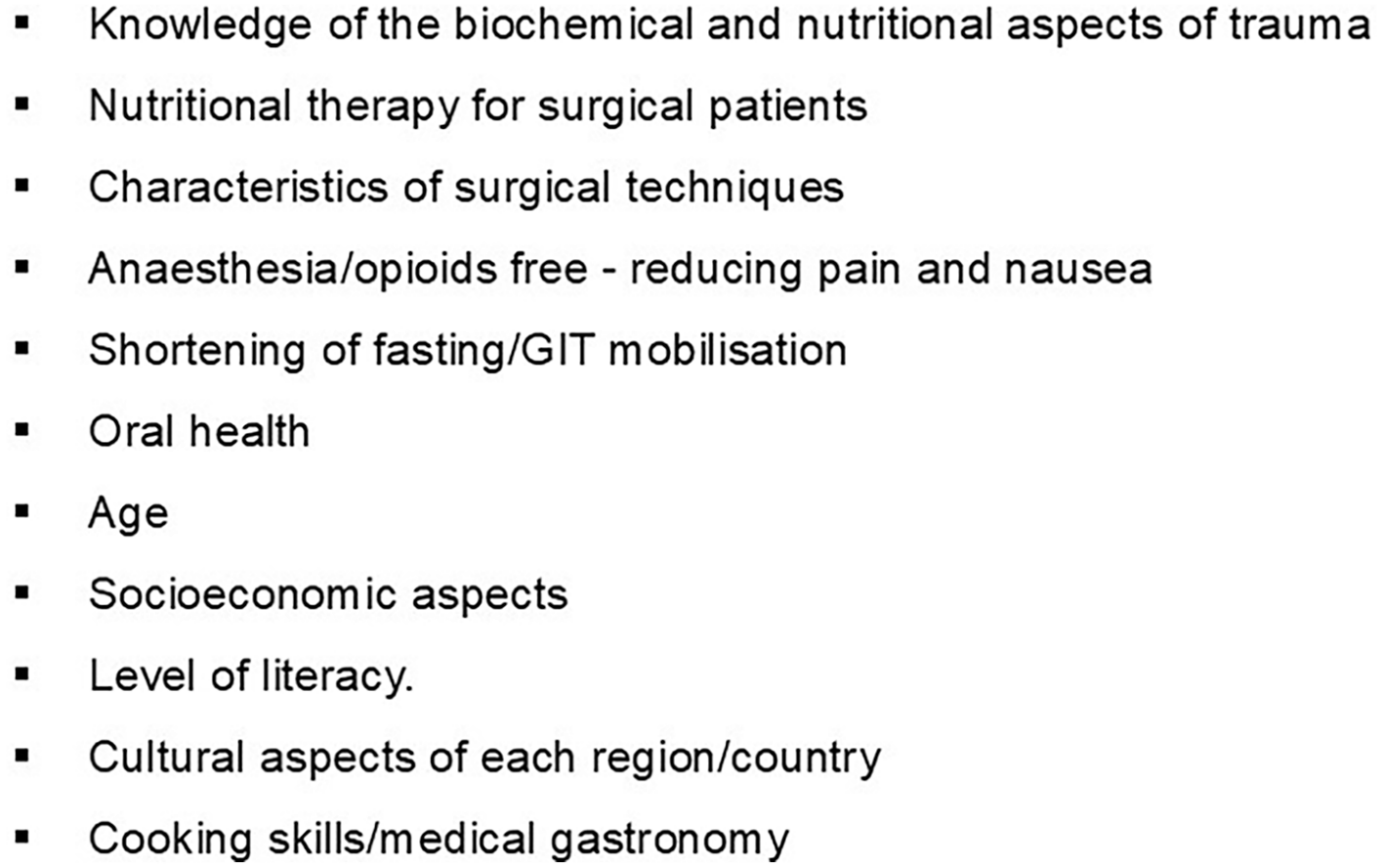
Determinants of diagnosis and nutritional intervention. In the early postoperative period following bariatric and metabolic surgery, by authors.

There is consensus on the importance of follow-up by a nutritionist specialized in metabolic surgery and clinical nutrition, given the presence of key variables that influence diagnosis and nutritional intervention in the early postoperative period (Figure 3).

It is important to emphasize that nutritional intervention should be systematic and individualized, as patients often present with signs and symptoms of qualitative malnutrition, reflecting the progressive and recurrent nature of metabolic disease.

In transit bipartition, episodes of diarrhea may occur due to accelerated intestinal transit, anatomical or functional limitation of duodenal and jejunal flow (3), or when the gastroileal anastomosis exceeds 4 cm in diameter, resulting in a greater proportion of nutrients being diverted to the ileal bridge and the relatively short 250 cm common limb. In such cases, the physiological effect may resemble that observed in biliopancreatic diversion with duodenal switch (BPD-DS), leading to the expected gastrointestinal side effects.

In the present case, no episodes of diarrhea or other intestinal adverse effects were observed during the first 30 postoperative days.

Santoro et al. (2) reported that mild constipation was rarely observed in their study. Most patients experienced an increase in bowel movement frequency, similar that observed prior to intestinal transit division. Additionally, the authors described softer stool consistency, accompanied by increased flatulence and stool odor.

However, these symptoms were rarely perceived as problematic by patients, who recognized that their occurrence was often associated with the intake of fatty foods. It is important to note that intestinal and dietary adaptation may occur over a period of up to two years following ileal surgical techniques that are not primarily mechanically restrictive. This adaptive process should be considered when evaluating patients, establishing nutritional diagnoses, and defining appropriate nutritional interventions.

It is important to recognize that inappropriate nutritional interventions, particularly those involving overly restrictive and prolonged dietary limitations, can negatively affect nutritional status and quality of life in these patients. Moreover, such approaches may compromise overall health outcomes, remission of metabolic diseases, and long-term weight maintenance. Surgical treatment of severe obesity represents an opportunity for meaningful lifestyle change through comprehensive nutrition education and the adoption of sustainable, healthy eating habits and behaviors.

The proposed nutritional protocol—characterized by early dietary introduction, structured weekly progression in food consistency, and systematic protein, vitamin, and mineral supplementation—proved to be safe and effective. This approach facilitated excellent food tolerance and normal intestinal function, with no gastrointestinal adverse effects observed during the early postoperative period following transit bipartition.

The limitations of this case report are inherent to its methodological design and preclude generalization of the findings to all patients undergoing transit bipartition. Accordingly, prospective studies, including randomized controlled trials with representative populations, are needed to provide robust evidence on early nutritional interventions and their impact on long-term outcomes.

## Patient perspective

“I’m extremely satisfied with my current dietary intake. I gradually introduced foods according to the prescribed plan and took care to chew thoroughly, including liquids, and to consume small amounts at each meal. I use a teaspoon or dessert spoon, avoid distractions, and always sit-down during meals. I can able to eat all foods as prescribed and have not experienced any episodes of vomiting or regurgitation during the first 30 days after surgery.”

## Data Availability

The datasets presented in this article are not readily available due to ethical restrictions to ensure patient privacy. Requests to access the datasets should be directed to the corresponding author, rossonicarina@gmail.com, Carina Rossoni.
